# Timing of surgery in professional baseball—an analysis of Major League Baseball pitchers who underwent ulnar collateral ligament reconstruction

**DOI:** 10.1016/j.xrrt.2025.100599

**Published:** 2025-11-07

**Authors:** Keigo Honoki, Lawrence Wengle, Timothy A. Burkhart, Scott Peters, John Theodoropoulos

**Affiliations:** a University of Toronto, University of Toronto Orthopaedic Sports Medicine, Toronto, Ontario, Canada; d Dovigi Orthopaedic Sports Medicine Clinic, Mount Sinai Hospital, Toronto, Ontario, Canada; b Kinesiology and Physical Education, University of Toronto, Toronto, Ontario, Canada; c Toronto Blue Jays Baseball Club, Toronto, Ontario, Canada

**Keywords:** Tommy John surgery, Ulnar collateral ligament reconstruction, Major league baseball, Return to play

## Abstract

**Background:**

The rates of return to the same level of play (RTSP), time to RTSP in Major League Baseball (MLB) pitchers, and missed days of the season after ulnar collateral ligament reconstruction (UCLR) have been reported previously. However, previous studies have shown a large disparity between time to RTSP and missed playing time during the season after UCLR. Additionally, the literature has not investigated the relation between the timing of UCLR during a season and the time to RTSP. The purpose of this study was to investigate how the timing of UCLR during the season affects the time it takes to RTSP.

**Methods:**

The data of MLB pitchers who underwent UCLR between January 2012 and December 2022 were obtained from publicly available online records. The rate of RTSP, the rate of RTSP in the next season, and the time to RTSP at the MLB level were collected. The timing of surgery performed during the season was grouped into 3 categories: early season (January to April), middle season (May to August), and late season (September to December). The rate of RTSP and time to RTSP were compared across each category.

**Results:**

Two hundred seventy-two MLB pitchers who underwent UCLR were included. Two hundred twelve pitchers successfully returned to MLB. Overall, the rate of RTSP after UCLR in MLB pitchers was 77.9% without any statistical difference among the 3 timing categories (*P* = .45). The rate of RTSP in the next MLB season after UCLR was 56.7% in the early season, 37.0% in the middle season, and 4.2% in the late season. Time to RTSP was 19.0 ± 7.2 months in the early season group, 20.7 ± 10.0 months in the middle season group, and 23.0 ± 8.6 months in the late season group. Time to RTSP in the early season group was significantly faster than in the late season group (*P* < .001).

**Conclusion:**

The timing of UCLR during the season has a significant effect on the absolute time to RTSP in MLB pitchers. Those who undergo surgery later in the season have significantly longer absolute time to RTSP than those earlier in the season.

Ulnar collateral ligament reconstruction (UCLR), known as Tommy John surgery, has been commonly performed for injuries to the ulnar collateral ligament (UCL) in Major League Baseball (MLB) pitchers.^3^^,^^4^^,^^6^^,^^8^ It has been reported that approximately 25% of MLB pitchers undergo UCLR at some point in their careers.^4^ As UCL injuries and UCLR are reported as the leading causes in time lost playing professional baseball, a better understanding of return to sport after UCLR is important in treating UCL injuries in MLB pitchers.^17^

Return to the same level of play (RTSP) at the MLB level after UCLR has been variably reported, ranging from 71 to 87%.^3^^,^^4^^,^^10^, ^11^, ^12^, ^13^, ^14^ Several previous reports found that the mean time to RTSP at the MLB level was between 17.0 and 20.5 months for MLB pitchers.^8^, ^9^, ^10^, ^11^, ^12^ Furthermore, a recent report demonstrated that pitchers undergoing UCLR missed an average of 180.2 days (5.9 months) of the MLB season. This showed a large disparity from the previously reported time from surgery to RTSP at the MLB level.^15^ We believe this disparity may be heavily influenced by the timing of surgery during the MLB season. However, the effects of the timing of UCLR in a season on the rate of RTSP at the MLB level and the time from UCLR to RTSP at the MLB level have not been previously investigated.

The purpose of this study was to investigate how the timing of UCLR during the season affects the rate of RTSP and the time it takes to RTSP in MLB pitchers. Ultimately, the results of this study can allow MLB staff and teams to make roster decisions to better plan the management of player personnel in the next season when any pitcher in the team undergoes UCLR. We hypothesize that the rate of RTSP is the same regardless of the timing of surgery. The timing of surgery during a season affects the time to RTSP. More specifically, having UCLR later in the season takes longer to reach RTSP than undergoing UCLR earlier in the season.

## Materials and methods

### Data collection

This retrospective cohort study used publicly available data, and a formal institutional review board was not required. Many previous MLB-related reports used publicly available online data.^1^^,^^2^^,^^7^^,^^9^^,^^10^^,^^13^, ^14^, ^15^ In this study, we obtained comprehensive data on MLB pitchers who had undergone UCLR from the same publicly available database^16^ previously used in multiple studies.^1^^,^^2^

MLB pitchers who played their last game at the MLB level prior to undergoing UCLR and whose UCLR was between January 2012 and December 2022 were included in this study. Position players and minor league pitchers were excluded. From the database, the month of UCLR, RTSP to the MLB level, and time to RTSP to the MLB level of each player who had UCLR were extracted. RTSP to the MLB level was defined as pitching in ≧1 game with an MLB team. Time to RTSP at the MLB level after UCLR was defined by the date from surgery to the date of the pitcher's return to pitching in ≧1 game with an MLB team.^15^^,^^16^ As there was a small number of cases in November, December, January, and February, for statistical purposes, the timing of the surgery in a season was grouped into 3 categories: early season (January to April), middle season (May to August), and late season (September to December). The rate of RTSP after UCLR was calculated in each group. Time to RTSP at the MLB level was also calculated in those who were able to return to RTSP at the MLB level.

### Statistics

All statistical analysis was performed using R version 4.0.3 software (R Foundation for Statistical Computing, Vienna, Austria) and R Commander version 1.54 (John Fox, McMaster University, Hamilton, ON, Canada). Fisher's exact test was performed to analyze any difference in RTSP rate among each group. Nonparametric Kruskal-Wallis testing was used to detect any significant difference between the 3 groups in terms of RTSP at the MLB level. Steel-Dwass testing was conducted between each group after Kruskal-Wallis testing. All *P* values were two-sided, and *P* values of .05 or less were considered statistically significant.

## Results

**Figure 1:**
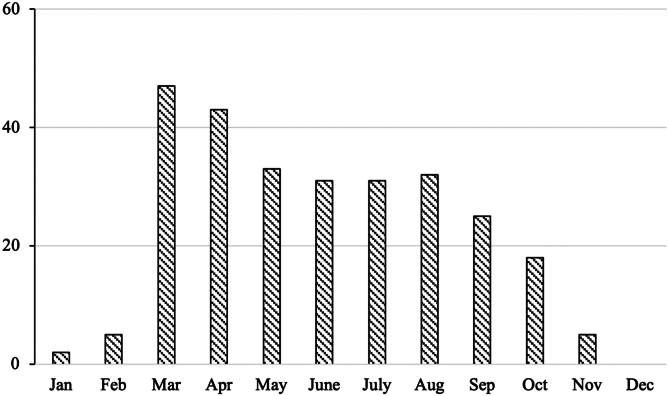
The distribution of UCLR timing by month in MLB pitchers between January 2012 and December 2022 (n = 272). *UCLR*, ulnar collateral ligament reconstruction; *MLB*, Major League Baseball.

Two hundred seventy-two MLB pitchers underwent UCLR between January 2012 and December 2022. The mean age was 27.5 ± 3.4 years. There were 201 right-sided pitchers and 71 left-sided pitchers. The trend of surgical timing of UCLR in MLB pitchers between 2012 and 2022 is shown in Figure 1. From November to February, fewer than 5 cases, respectively, were performed each month, whereas UCLR was most frequently performed in March and April.

The rate of RTSP, the rate of RTSP in the next season, and the time to RTSP at the MLB level are shown in Table I. There was no significant difference in the RTSP rate among each group (*P* = .45). Kruskal-Wallis testing among 3 groups in time to RTSP was significant (*P* = .0075). Comparison between each group (early season versus middle season, early season versus late season, and middle season versus late season) revealed a significant difference in time to RTSP between the early and late season groups. There was significantly faster time to RTSP in the early season cohort compared to the late season group (*P* < .001). Early versus middle season and middle season versus late season did not significantly differ (*P* = .923 and *P* = .181, respectively).

**Table I:** The rate of RTSP (%), RTSP in the next year (%), and time to RTSP (months) at the MLB level based on the timing of the UCLR during the season.

Timing	n	RTSP (%)	RTSP in the next season (%)	Time to RTSP (months)
Early (January-April)	97	74.2	56.7	19.0 ± 7.2
Middle (May-August)	127	82.1	37.0	20.7 ± 10.0
Late (September-December)	48	77.1	4.2	23.0 ± 8.6
Total (n, %, months)	272	77.9	38.2	20.5 ± 9.0

*RTSP*, return to same level of play; *MLB*, Major League Baseball; *UCLR*, ulnar collateral ligament reconstruction.

Data are expressed as the number of players (n), percentage (%) for the RTSP rate, the months for time to RTSP.

## Discussion

This study reported the timing of UCLR in MLB pitchers between 2012 and 2022. It demonstrated that UCLR was less frequently performed in the off-season between November and February and more commonly during the early season in March and April. DeFroda et al^5^ reported that 62% of UCL tears occurred in the first three months of the season (April–June). These results suggest that pitchers may not feel elbow symptoms in the off-season because of the lighter workload on their elbows. Pitchers tend to have UCLR earlier in the season than in the late season, but more research is required to characterize the reasons for this.

Previous studies reported RTSP after UCLR in MLB pitchers ranging from 71 to 87%.^3^^,^^4^^,^^10^, ^11^, ^12^, ^13^, ^14^ Our data showed an RTSP rate of 77.9% in MLB pitchers, which is comparable to the literature. There was no significant difference in the rate of RTSP in MLB pitchers in the early, middle, and late seasons. The rate of RTSP in the following MLB season after UCLR was also investigated in the present study, which was 56.7% in the early season, 37.0% in the middle season, and 4.2% in the late season. This result indicated that only around half of pitchers having UCLR before the end of April returned to MLB in the next season, and those having UCLR in the middle to late season had a further low rate of RTSP in the next season.

With regard to time to RTSP, Erickson et al^9^ described an average of 20.5 months of RTSP after UCL reconstruction in MLB pitchers. This group^8^ also reported in a separate study that the average time to RTSP after UCLR was 17.3 ± 13.0 months in professional baseball pitchers. Gibson et al^10^ reported that MLB pitchers returned to the same level of play at the MLB level at a mean of 18.5 months after UCLR. Jiang et al^12^ reported that the time to RTSP in MLB pitchers was 17.1 months. Conversely, the recent report by Meldau et al^15^ revealed that pitchers undergoing UCLR missed 5.9 months of the MLB season, demonstrating a significant disparity from the previously reported time from surgery to RTSP at the MLB level. Previous studies investigating RTSP after UCLR did not consider the timing of surgery during the season; we believe the disparity in time to return to play may be attributable to the timing of surgery performed during the calendar year. The results of our study confirm that UCLR during the early season provided significantly faster RTSP compared to late season. We believe that pitchers who undergo UCLR early in the MLB season can optimize their timing to return the following season if they make a good surgical recovery. The most important part of this study is that this information would also allow MLB staff to make roster decisions to better plan the management of player personnel in the upcoming season. For example, if MLB staff and teams can expect a pitcher having UCLR back to the team before the All-Star Break or trade deadline, they may not have to sign an extra pitcher in the offseason. As such, the results of this study are practical for MLB staff and teams. The results of the study also impact the measures in the research about UCLR. We believe that utilizing time to RTSP in the MLB to compare the clinical outcomes and recovery of UCL reconstruction incompletely evaluates this patient population. This study has demonstrated that the specific timing of UCLR in a season significantly affects the time to RTSP in the MLB. We suggest that researchers utilize missed days or missed playing time during the regular season instead of absolute time to RTSP or match the timing of the UCLR in a season in the cohort when comparing to better represent the recovery after UCLR in professional baseball players, as timing of the season can be a confounding factor of time to RTSP. Further research that can confirm the relation between time to RTSP and missed days of the season should be performed in the future.

The present study is not without limitations. First, publicly published online data were utilized in our study. Any pitcher not included in this public database may have been excluded from the present study. However, we followed methods similar to those of other studies using the same database.^1^^,^^2^ Second, UCLR was performed by many different surgeons throughout this study. The details and variances in surgical techniques were not provided. However, UCL repair was differentiated in the database, and only UCL reconstruction was included in this study. Third, some detailed data of each player, such as dominance and type of pitcher (starter or reliever), were not described in the present study. Fourth, other factors we did not take into account in this study may affect the return to play at the MLB other than elbow condition, competence, and performance. Those include social factors including roster management or contractual concerns. Lastly, because there were so few cases in November, December, January, and February, we categorized the months into 3 groups (early, middle, and late season). However, this categorization can include selection and publication bias. Although more research with more reliable data is required to further support our results, the results of the present study will still be important information for team doctors, players, and teams for the decision-making regarding the management of UCL injury.

## Conclusions

The timing of UCLR significantly affects the absolute time to RTSP in MLB pitchers. More than half of pitchers having UCLR in the early season returned to the MLB in the following season. Those who undergo surgery later in the season have a significantly longer time to RTSP than those earlier in the season.

## Disclaimers:

Funding: No funding was disclosed by the authors.

Conflicts of interest: The authors, their immediate families, and any research foundations with which they are affiliated have not received any financial payments or other benefits from any commercial entity related to the subject of this article.
